# Supplementary material for: A comparative analysis of dementia strategies of seventeen European Countries in the context of Glasgow Declaration and WHO’s Global Action Plan
Source: PLoS One. 2025 Nov 12;20(11):e0319722. doi: 10.1371/journal.pone.0319722 (PMC12611155; doi:10.1371/journal.pone.0319722)
Supplement: S1 Text — (DOCX) [file pone.0319722.s001.docx]

**Appendix – A: Methodological Note**

After data cleaning, the following process is undertaken to make the text usable for analysis; this is called pre-processing of the text (1,2):

**Tokenising:** Breaking the words into smaller fragments: sentences or words. For determining the optimal number of clusters (k) and undertaking cluster analysis, the text is broken down into sentences, technically called “sentence tokenised”. For all further analysis, the text is “word tokenised”.

**Part-of-Speech Tagging (POS-Tagging):** Identifying whether the word is a noun, verb, adjective etc. This is done to ensure the correct contextualisation of the words.

**Lemmatising:** This process reduces the words to their roots. For example, converting “cared”, “caring”, “cares” and “carer”, to the root, “to care”. Lemmatising is done after POS-tagging to make sure that nouns, sounding like verbs, do not get converted to their roots.

**Lower Casing:** The process of converting all the words into lowercase. Since, upper case and lowercase letters are interpreted differently at binary level, it is advisable to convert all the words into lower case so that, “Care”, “CARE” and “care” are not counted as different words. This is preferably done after lemmatising.

**Stopwords removal:** In the NLP parlance, the most frequently occurring words, yet not contributing to the meaning of the text, are called stopwords. For example, “the”, “to”, “also”. If not removed, they would dominate the word cloud and further analysis.

**Removal of punctuation, numbers and special characters:** Depending upon the nature of analysis, punctuations be removed (or retained). Our analysis does not require these and therefore, we have removed these. If the text is extracted through html or pdf, it is likely to have extra spaces too. We removed all extra spaces as well, at this juncture.

The text is now, now ready to be used for further analysis.

**References**

1. Germec M. Text preprocessing with Natural Language Processing (NLP) [Internet]. LinkedIn. 2023 [cited 2024 Apr 20]. Available from: https://www.linkedin.com/pulse/text-preprocessing-natural-language-processing-nlp-germec-phd

2. Harshith. Text Preprocessing in Natural Language Processing: Significance of text preprocessing in the performance of models [Internet]. Towards Data Science. 2019 [cited 2024 Apr 20]. Available from: https://towardsdatascience.com/text-preprocessing-in-natural-language-processing-using-python-6113ff5decd8
