# Supplementary material for: A comparative analysis of dementia strategies of seventeen European Countries in the context of Glasgow Declaration and WHO’s Global Action Plan
Source: PLoS One. 2025 Nov 12;20(11):e0319722. doi: 10.1371/journal.pone.0319722 (PMC12611155; doi:10.1371/journal.pone.0319722)
Supplement: S1 Table — (DOCX) [file pone.0319722.s002.docx]

Table s1: Countries by Cluster Membership, Dementia Prevalence, Demographic Profile, per Capita GDP and Predominant Healthcare Financing Model

| **Countries**  **(Year^1^ of Latest Dementia Strategy)** | **Dementia Prevalence (percentage of total population)** (1)**^, 5^** | **Population**(2)**^, 6^** | **Share of Population above 65 years of age (%)**(2)**^, 6^** | **Life Expectancy at Birth**  **(Years)** (2)**^, 6^** | **Healthcare Financing Model (Predominant)** (3)**^, 7^** | **GDP per capita PPP**  **(EUR)** (4)**^, 6^** |
| --- | --- | --- | --- | --- | --- | --- |
| **Cluster 1** | | | | | | |
| Austria  (2015) | 1.66 | 8922082 | 19.44 | 81.58 | Bismarck | 53817.31 |
| Netherlands  (2021) | 1.49 | 17501696 | 19.95 | 81.69 | Bismarck + private voluntary insurance | 57334.7 |
| Israel  (2013) | 0.94 | 8900059 | 11.93 | 82.26 | Bismarck | 42379.16 |
| Scotland  (2023)^8^ |  |  |  |  |  |  |
| Gibraltar^2,3^  (2018) | NA | 32670 | 20.43 | 79.33 | Beveridge |  |
| Wales^8^  (2018) |  |  |  |  |  |  |
| Denmark  (2017) | 1.51 | 5854241 | 20.27 | 81.38 | Beveridge | 58802.96 |
| Cyprus  (2012) | 1.17 | 1244188 | 14.48 | 81.20 | Bismarck + Beveridge | 43416.97 |
| Belgium (Flanders)  (2016) | 1.69 | 11611420 | 19.42 | 81.88 | Bismarck | 52174.91 |
| Finland  (2012) | 1.74 | 5535992 | 22.89 | 82.04 | Beveridge | 48614.82 |
| **Cluster 2** | | | | | | |
| Germany  (2020) | 1.91 | 83408555 | 22.17 | 80.63 | Bismarck | 53395.65 |
| **Cluster 3** | | | | | | |
| Norway^4^  (2021) | 1.41 | 5403021 | 18.10 | 83.23 | Beveridge | 65915.53 |
| **Cluster 4** | | | | | | |
| England^8^  (2020) |  |  |  |  |  |  |
| Ireland  (2014) | 1.09 | 4986526 | 14.83 | 82.00 | Beveridge | 104671.9 |
| Malta  (2015) | 1.38 | 526748 | 18.87 | 83.78 | Beveridge | 46598.64 |
| Northern Ireland^8^  (2011) |  |  |  |  |  |  |
| Greece  (2015) | 1.99 | 10445365 | 22.51 | 80.11 | Bismarck | 29630.93 |
|  | | | | | | |
| United Kingdom | 1.56 | 67281040 | 18.92 | 80.74 | Beveridge | 45567.57 |

**Notes:** 1. Some countries have revised their dementia strategies at regular intervals. The year mentioned here represents the year of the most recently revised dementia strategy of each of these countries.

2. Gibraltar is a British Overseas Territory and left the EU in 2016. It is a signatory to the Glasgow Declaration 2014 and is a member of Alzheimer’s Europe.

3. Gibraltar launched its latest Dementia Strategy in May 2023, but it is still not available on Alzheimer’s Europe’s website (from where all strategies are downloaded).

4. Norway is not a member of the EU.

5. Estimated Prevalence in 2018.

6. These are 2021 figures.

7. Compulsory Social Insurance (Bismarck Model), General Taxation (Beveridge Model).

8. Data on all the indicators are available for the United Kingdom, and not for separate constituent countries.

**References**

1. Alzheimer Europe. Glasgow Declaration [Internet]. 2014 [cited 2023 Oct 31]. Available from: https://www.alzheimer-europe.org/policy/campaign/glasgow-declaration-2014#:~:text=The%20Glasgow%20Declaration%20called%20for,global%20action%20plan%20on%20dementia.

2. United Nations, Department of Economic and Social Affairs, Population Division. World Population Prospects: The 2022 Revision [Internet]. custom data acquired via website; 2022 [cited 2024 Apr 18]. Available from: https://population.un.org/dataportal/

3. OECD/European Observatory on Health Systems and Policies. Country Health Profile 2023 [Internet]. OECD Publishing, Paris/European Observatory on Health Systems and Policies, Brussels; 2023 [cited 2024 Apr 9]. (State of Health in the EU). Available from: https://health.ec.europa.eu/state-health-eu/country-health-profiles_en

4. World Bank. GDP per capita, PPP (constant 2017 international $) [Dataset] [Internet]. World Development Indicators; 2024. Available from: https://data.worldbank.org/indicator/NY.GDP.PCAP.PP.KD
