# Supplementary material for: A comparative analysis of dementia strategies of seventeen European Countries in the context of Glasgow Declaration and WHO’s Global Action Plan
Source: PLoS One. 2025 Nov 12;20(11):e0319722. doi: 10.1371/journal.pone.0319722 (PMC12611155; doi:10.1371/journal.pone.0319722)
Supplement: S2 Table — (DOCX) [file pone.0319722.s003.docx]

Table s2: Bigrams’ Frequencies of Clusterwise Dementia Strategy Text

| **Bigram** | **Frequency** | **Combined Frequency** |
| --- | --- | --- |
| Cluster 1 | | |
| ('health', 'care') | 110 | 582 |
| ('care', 'support') | 85 |  |
| ('care', 'service') | 53 |  |
| ('support', 'care') | 51 |  |
| ('support', 'people') | 45 |  |
| ('social', 'care') | 41 |  |
| ('care', 'people') | 40 |  |
| ('care', 'home') | 39 |  |
| ('care', 'patient') | 34 |  |
| ('care', 'partner') | 30 |  |
| ('informal', 'caregiver') | 27 |  |
| ('social', 'service') | 27 |  |
| ('people', 'live') | 172 | 266 |
| ('people', 'family') | 31 |  |
| ('family', 'friend') | 63 |  |
| ('dementiarelated', 'impairment') | 73 | 150 |
| ('impairment', 'family') | 28 |  |
| ('quality', 'life') | 49 |  |
| Cluster 2 | | |
| ('long-term', 'care') | 178 | 684 |
| ('family', 'caregiver') | 91 |  |
| ('care', 'people') | 83 |  |
| ('nursing', 'care') | 52 |  |
| ('care', 'support') | 46 |  |
| ('care', 'facility') | 45 |  |
| ('care', 'service') | 40 |  |
| ('palliative', 'care') | 35 |  |
| ('care', 'need') | 30 |  |
| ('short-term', 'care') | 28 |  |
| ('care', 'home') | 28 |  |
| ('nurse', 'care') | 28 |  |
| ('federal', 'state') | 84 | 222 |
| ('national', 'strategy') | 82 |  |
| ('care', 'insurance') | 38 |  |
| ('local', 'authority') | 38 |  |
| ('welfare', 'organisation') | 32 |  |
| ('authority', 'association') | 32 |  |
| ('health', 'service') | 111 | 207 |
| ('service', 'people') | 39 |  |
| ('counselling', 'service') | 29 |  |
| ('provide', 'information') | 28 |  |
| Cluster 3 | | |
| ('care', 'service') | 126 | 387 |
| ('health', 'care') | 110 |  |
| ('health', 'service') | 32 |  |
| ('longterm', 'care') | 31 |  |
| ('personcentred', 'care') | 26 |  |
| ('respite', 'care') | 22 |  |
| ('care', 'facility') | 21 |  |
| ('service', 'people') | 19 |  |
| ('family', 'member') | 110 | 151 |
| ('individual', 'family') | 22 |  |
| ('user', 'family') | 19 |  |
| Cluster 4 | | |
| ('social', 'care') | 117 | 845 |
| ('care', 'people') | 79 |  |
| ('care', 'support') | 73 |  |
| ('care', 'home') | 69 |  |
| ('care', 'service') | 64 |  |
| ('palliative', 'care') | 61 |  |
| ('people', 'carers') | 51 |  |
| ('primary', 'care') | 48 |  |
| ('health', 'care') | 46 |  |
| ('individual', 'caregiver') | 45 |  |
| ('support', 'people') | 44 |  |
| ('day', 'care') | 38 |  |
| ('family', 'carers') | 37 |  |
| ('healthcare', 'professional') | 37 |  |
| ('management', 'care') | 36 |  |
| ('health', 'social') | 93 | 203 |
| ('health', 'service') | 57 |  |
| ('public', 'health') | 53 |  |
