# Supplementary material for: A comparative analysis of dementia strategies of seventeen European Countries in the context of Glasgow Declaration and WHO’s Global Action Plan
Source: PLoS One. 2025 Nov 12;20(11):e0319722. doi: 10.1371/journal.pone.0319722 (PMC12611155; doi:10.1371/journal.pone.0319722)
Supplement: S3 Table — (DOCX) [file pone.0319722.s004.docx]

Table s3: Trigrams’ Frequencies of Clusterwise Dementia Strategy Text

| **Trigram** | **Frequency** | **Combined Frequency** |
| --- | --- | --- |
| Cluster 1 | | |
| ('post', 'diagnostic', 'support') | 41 |  |
| ('health', 'social', 'care') | 23 |  |
| ('health', 'care', 'service') | 20 |  |
| ('health', 'care', 'social') | 17 |  |
| ('support', 'people', 'live') | 17 |  |
| ('care', 'service', 'social') | 15 |  |
| ('service', 'social', 'service') | 15 |  |
| ('people', 'live', 'care') | 15 |  |
| ('care', 'partners unpaid', 'carers') | 14 | 177 |
| ('people', 'dementia-related', 'impairment') | 70 |  |
| ('impairment', 'family', 'friend') | 27 |  |
| ('dementia-related', 'impairment', 'family') | 26 | 123 |
| Cluster 2 | | |
| ('longterm', 'care', 'insurance') | 36 |  |
| ('longterm', 'care', 'facility') | 23 |  |
| ('residential', 'longterm', 'care') | 21 |  |
| ('fully', 'residential', 'longterm') | 20 |  |
| ('health', 'longterm', 'care') | 16 |  |
| ('longterm', 'care', 'counsel') | 16 |  |
| ('longterm', 'care', 'service') | 15 | 147 |
| ('health', 'service', 'research') | 25 |  |
| ('hospice', 'palliative', 'care') | 22 |  |
| ('health', 'service', 'people') | 16 |  |
| ('definition', 'care', 'need') | 15 | 78 |
| Cluster 3 | | |
| ('health', 'care', 'service') | 78 |  |
| ('specialist', 'health', 'service') | 23 |  |
| ('municipal', 'health', 'care') | 21 |  |
| ('service', 'user', 'family') | 19 |  |
| ('longterm', 'care', 'facility') | 19 |  |
| ('home', 'care', 'service') | 18 |  |
| ('respite', 'care', 'service') | 17 |  |
| ('day', 'activity', 'service') | 16 |  |
| ('health', 'care', 'personnel') | 15 |  |
| ('patient', 'service', 'user') | 10 | 236 |
| ('norwegian', 'directorate', 'health') | 54 |  |
| ('national', 'professional', 'guideline') | 13 |  |
| ('national', 'advisory', 'unit') | 13 |  |
| ('norwegian', 'health', 'association') | 12 |  |
| ('competency', 'enhancement', 'plan') | 12 |  |
| ('norwegian', 'national', 'advisory') | 11 | 115 |
| Cluster 4 | | |
| ('health', 'social', 'care') | 82 |  |
| ('health', 'service', 'executive') | 41 |  |
| ('caregiver', 'family', 'member') | 30 |  |
| ('social', 'care', 'service') | 19 |  |
| ('individual', 'caregiver', 'family') | 19 |  |
| ('end', 'life', 'care') | 18 |  |
| ('day', 'care', 'centers') | 18 |  |
| ('social', 'care', 'staff') | 15 |  |
| ('hospital', 'care', 'home') | 12 |  |
| ('people', 'family', 'carers') | 12 |  |
| ('social', 'care', 'professional') | 11 | 277 |
