# Supplementary material for: A comparative analysis of dementia strategies of seventeen European Countries in the context of Glasgow Declaration and WHO’s Global Action Plan
Source: PLoS One. 2025 Nov 12;20(11):e0319722. doi: 10.1371/journal.pone.0319722 (PMC12611155; doi:10.1371/journal.pone.0319722)
Supplement: Attachment — Submitted filename: Response to Reviewers.docx [file pone.0319722.s006.docx]

**04 May 2025**

**To**

**Dr Surangi Jayakodi**

*Academic Editor*

PLOS One

**Dear Dr Surangi Jayakodi,**

I am pleased to learn you found the topic of our manuscript important. I would like to thank you and the reviewers for carefully considering my article and sparing your valuable time to give insightful comments. I am submitting herewith, the revised manuscript (marked up version, edited with track changes, as well as the unmarked version). I have worked on the comments from the editor as well as the Reviewer 1 and have tried my best to comply with the journal requirements, as recommended. The responses to each of the reviewer’s comments / journal recommendations are as under:

**Editorial Comments:**

**Comment 1:**

There should be a clear flow of information in the manuscript, starting from the introduction, justification for the current project, objectives, methodology etc. Although the authors mentioned the subtopics accordingly, the information flow is lacking. Information presented in a mixed manner which affects the readability and understanding.

**Response 1:**

The manuscript is restructured to ensure the clear flow of information, and it is ensured that there are no overlaps in introduction, justification for the current project, objectives, methodology, results and discussion sections.

**Comment 2:**

Most of the article description have used first-person pronoun ‘We’. Using first-person language may detract from the arguments and/or viewpoints present in the writing. Therefore, avoid using first-person frequently, you may use passive voice instead

Eg. ‘*We apply k-means cluster analysis to classify the NDSs based on similarities’*

Suggest rewording as follows.

NDSs based on similarities were classified using k-means cluster analysis.

**Response 2:**

Sentences that were in active voice / first-person pronoun in the original draft are transformed into passive voice, at all instances in the manuscript.

**Response to the Comments of Reviewer 1**

**Comment 1:**

The authors have attempted to investigate the important issue of how well the national dementia strategies (NDSs) of 15 European countries align with the Glasgow Declaration and the WHO’s Global Action Plan guidance. However, the current version of the article does not meet academic standards, as information relevant to the introduction, methods, and results is interwoven across all sections. For example, the introduction includes methodological details, and vice versa. I strongly recommend that the authors carefully revise the text to ensure that each section is structured appropriately.

**Response 1:**

The manuscript is restructured to ensure that there are no overlaps of contents across sections, and each section contains the text relevant to the said section. The redundancies that have arisen because of the overlap in the original draft are also removed.

Additionally, Objectives of the study are now, explicitly stated in the subsection of the Methods section

**Comment 2:**

Although the primary objective of the study is to examine how well the selected NDSs comply with the Glasgow Declaration and the WHO’s Global Action Plan, the article does not directly compare the NDSs with these benchmark criteria. Furthermore, the discussion is not structured around the key action areas outlined in the WHO framework or the Glasgow Declaration.

**Response 2:**

Added two sub-sections, in the Discussion section, to highlight the extent of adherence of NDSs to the fundamental rights of Glasgow Declaration and the key action areas of the WHO’s global action plan.

**Comment 3:**

It is also unclear why more than half of the discussion focuses on the costs and cost-effectiveness of dementia interventions, given that these were not the primary focus of the study.

**Response 3:**

The discussion on cost-effectiveness of the provisions in the national dementia strategies is abridged and put in perspective of the NDSs’ compliance with Glasgow Declaration and WHO’s Global Action Plan.

**Comment 4:**

Additionally, a clear summary of findings is missing, and the results are not interpreted in relation to the WHO key action areas or the Glasgow Declaration.

**Response 4:**

Conclusion section is revised to present the summary of findings.

**Additionally**

A section on Limitations of this Study is added, which was discussed as a part of the Discussion section in the original draft.

Abstract is edited to reflect the changes made in the manuscript.

**Journal Requirements**

**Comment 1:**

Please ensure that your manuscript meets PLOS ONE's style requirements, including those for file naming. The PLOS ONE style templates can be found at <https://journals.plos.org/plosone/s/file?id=wjVg/PLOSOne_formatting_sample_main_body.pdf> and <https://journals.plos.org/plosone/s/file?id=ba62/PLOSOne_formatting_sample_title_authors_affiliations.pdf>

**Response 1:**

Title page is now, formatted as per the guidelines shared.

Headings are now, formatted as per the guidelines shared.

**Comment 2:**

We note that the grant information you provided in the ‘Funding Information’ and ‘Financial Disclosure’ sections do not match.

When you resubmit, please ensure that you provide the correct grant numbers for the awards you received for your study in the ‘Funding Information’ section.

**Response 2:**

There are no grant numbers associated with the funding. However, I have revised the funding statement as under:

**Revised Funding Statement**

Smruti Bulsari, University of Essex, is supported by the National Institute for Health and Care Research – Applied Research Collaboration East of England (NIHR ARC EoE) and the Alzheimer’s Society, funded through a Post-Doctoral Fellowship. The NIHR ARC EoE is hosted by Cambridgeshire and Peterborough NHS Foundation Trust. The views expressed are those of the authors and not necessarily those of the NIHR or the Department of Health and Social Care.

**Comment 3:**

Thank you for stating the following in the Competing Interests section:

I have read the journal's policy and the authors of this manuscript have the following competing interests: One of the co-authors (RK) is currently serving on the editorial board (academic editor) of PLOS One.

Please confirm that this does not alter your adherence to all PLOS ONE policies on sharing data and materials, by including the following statement: "This does not alter our adherence to PLOS ONE policies on sharing data and materials.” (as detailed online in our guide for authors [http://journals.plos.org/plosone/s/competing-interests](https://linkprotect.cudasvc.com/url?a=http%3a%2f%2fjournals.plos.org%2fplosone%2fs%2fcompeting-interests&c=E,1,f6lSd0sWd6uBGtT9aC5hFKdWs2t6cvxgAPRAUCOLJWOVgytzYLX4eBCzdnIsp6fHWx3nPjwJGq6ztjeYMKXRRlPTSn4IH4zW5gPkd_IYsgtKc9SLI9fsdqgpxw,,&typo=1&ancr_add=1)).

**Response 3:**

**Updated Competing Interest Statement:**

I have read the journal's policy, and the authors of this manuscript have the following competing interests: One of the co-authors (RK) is currently serving on the editorial board (academic editor) of PLOS One. This does not alter our adherence to PLOS ONE policies on sharing data and materials.

**Comment 4:**

Thank you for stating the following financial disclosure:

This study is funded by National Institute of Health and Care Research (NIHR) Applied Research Collaboration (ARC) East of England and Alzheimer's Society

Please state what role the funders took in the study. If the funders had no role, please state: "The funders had no role in study design, data collection and analysis, decision to publish, or preparation of the manuscript."
If this statement is not correct you must amend it as needed.
Please include this amended Role of Funder statement in your cover letter; we will change the online submission form on your behalf.

**Response 4:**

**Updated Financial Disclosure**

This study is funded by National Institute of Health and Care Research (NIHR) Applied Research Collaboration (ARC) East of England and Alzheimer's Society. The funders had no role in study design, data collection and analysis, decision to publish, or preparation of the manuscript.

**Comment 5:**

Thank you for stating the following in the Acknowledgments Section of your manuscript:

Smruti Bulsari, University of Essex, is supported by the National Institute for Health and Care Research – Applied Research Collaboration East of England (NIHR ARC EoE) and the Alzheimer’s Society, funded through a Post-Doctoral Fellowship. The NIHR ARC EoE is hosted by Cambridgeshire and Peterborough NHS Foundation Trust. The views expressed are those of the authors and not necessarily those of the NIHR or the Department of Health and Social Care.

We note that you have provided funding information that is not currently declared in your Funding Statement. However, funding information should not appear in the Acknowledgments section or other areas of your manuscript. We will only publish funding information present in the Funding Statement section of the online submission form.
Please remove any funding-related text from the manuscript and let us know how you would like to update your Funding Statement.

**Response 5:**

Acknowledgement section is now removed, in the revised manuscript, and the Funding Statement is revised as in **response 2 (to comment 2) under the heading Journal requirements (above)**.

**Comment 6:**

[Please include captions for your Supporting Information files at the end of your manuscript, and update any in-text citations to match accordingly. Please see our Supporting Information guidelines for more information: http://journals.plos.org/plosone/s/supporting-information.](https://linkprotect.cudasvc.com/url?a=https%3a%2f%2fPlease%2520include%2520captions%2520for%2520your%2520Supporting%2520Information%2520files%2520at%2520the%2520end%2520of%2520your%2520manuscript%2c%2520and%2520update%2520any%2520in-text%2520citations%2520to%2520match%2520accordingly.%2520Please%2520see%2520our%2520Supporting%2520Information%2520guidelines%2520for%2520more%2520information%3a%2520http%3a%2fjournals.plos.org%2fplosone%2fs%2fsupporting-information.%2520&c=E,1,g-YndpiSqi8ypi71WldOoHb9FKJmVIeSByxFHgs89Ovk5C3sf9PU8uaaw3P0HzD7F96fUlcfpXTvWaoamIpTPjBXja5YIBuE5AJdUGbOyNHMrMCdiWyD&typo=1)

**Response 6:**

Created four separate files for supplementary information and details given along with caption and one-line title, at the end of the manuscript. In-text references are edited and collated accordingly.

**Finally, with reference to the recommendation** “While revising your submission, please upload your figure files to the Preflight Analysis and Conversion Engine (PACE) digital diagnostic tool, <https://pacev2.apexcovantage.com/>. PACE helps ensure that figures meet PLOS requirements.”

**Response:** Figure files uploaded to PACE V2.

Also, figures have been renamed in the manuscript, to match the renamed figures as per PLOS guidelines by PACE V2. All corresponding references to the figure in the manuscript are also renamed.

Thank you for your consideration of our work. We look forward to your response and to working with you through the review process.

Sincerely,

**Smruti Bulsari**

Senior Research Officer, University of Essex (UNITED KINGDOM):

e-mail: [smruti.bulsari@essex.ac.uk](mailto:smruti.bulsari@essex.ac.uk)
