# Supplementary material for: A comparative analysis of dementia strategies of seventeen European Countries in the context of Glasgow Declaration and WHO’s Global Action Plan
Source: PLoS One. 2025 Nov 12;20(11):e0319722. doi: 10.1371/journal.pone.0319722 (PMC12611155; doi:10.1371/journal.pone.0319722)
Supplement: Attachment — Submitted filename: Response to Reviewers_Ver2.0.docx [file pone.0319722.s007.docx]

**10 September 2025**

**To**

**Dr Surangi Jayakodi**

*Academic Editor*

PLOS One

**Dear Dr Surangi Jayakodi,**

I am indeed thankful to the reviewers for going through the revised manuscript and their kind, encouraging words regarding my revision. I also thank you and the reviewers for sparing your valuable time for the necessary co-ordination and giving finer comments on my revised draft. I am submitting herewith, the revised manuscript (marked up version, edited with track changes, as well as the unmarked version). I have worked on the comments of both the reviewers and the responses to each of the reviewer’s comments are as under:

**Response to the comments of Reviewer 1**

**Comment 1:** While the discussion now engages more meaningfully with the WHO and Glasgow benchmarks, an explicit comparison table or matrix would have added further clarity. Similarly, although the discussion on cost-effectiveness is now better contextualised, it remains relatively long compared to the main findings and could be further shortened.

**Regarding comparison matrix:** A matrix is developed and placed in the results section under the heading “Comparison of NDSs clusters with the Fundamental Rights of the Glasgow Declaration and the KAAs of the WHO’s Global Action Plan”, along with a brief explanation.

Because this section now makes a comparison of the NDSs with the fundamental rights of the Glasgow Declaration and the KAAs of the Global Action Plan, the titles of sub-section in the discussion section are changed as:

Revised Title: “NDSs Clusters in the context of the Fundamental Rights of the Glasgow Declaration”

Original Title: “Comparison of NDSs Clusters with the Fundamental Rights of the Glasgow Declaration”, and

Revised Title: “NDSs Clusters in the context of the Key Action Areas of WHO’s Global Action Plan”

Original Title: “Comparison of NDSs Clusters with the Key Action Areas of WHO’s Global Action Plan”

Text has been edited in these sub-sections of the discussion section to remove redundancies with the newly added section on Comparison of NDSs clusters with the “Fundamental Rights of the Glasgow Declaration and the KAAs of the WHO’s Global Action Plan”.

**Regarding the discussion on cost-effectiveness:** It is now shortened, while retaining its essence and attempting to make it even more contextual.

**Response to the comments of Reviewer 2**

**Comment 1:** Authors mentioned the objectives in the Methods section. The objectives should be at the end of the justification and before the “Methods” section

**Response 1:** The objectives are now, moved to the end of the justification.

**Comment 2:** It is better if the methods are described in a concise way which all readers can grab how the study is conducted clearly. Also, it is great if the authors mention, study period which helps the reader to understand by which year these strategic plans are considered for the study.

**Response 2:** Methods section is revised to be presented in a concise form.

Regarding study period, this text is added at the end of the second paragraph of “Data for this Study….” Section. The text explains why it is difficult to explicitly specify the time-period of this study: “…Therefore, this makes it difficult to explicitly specify the time-period of this study. Though, the oldest dementia strategy is of Cyprus, which is published in 2012 and the most recent is Germany’s, published in 2023; the dementia strategies used in this study were downloaded in November 2023; the year for the NDS of each of these countries are given in are Table s1”.

**Comment 3:** Table 1 (Page 21) depicts the cluster membership of countries based on NDSs contents. This is an output from the analysis by the authors for sure; According to this table, there are 17 countries categorized into clusters whereas for the study, there are only 15 countries included. How can the “Denmark” and “Greece” come into play additionally while the NDSs of those two countries are not included in the study?

**Response 3:** We are extremely sorry for this oversight. We rechecked the data and there are 17 countries. We have included the NDSs of Denmark and Greece in our analysis. Therefore, we have replaced 17 in place of 15 European countries, everywhere in the manuscript, abstract and the title. We rechecked and confirmed that in all our outputs, there are 17 European countries, which includes Greece and Denmark. Therefore, in the methods section too, we have added these two countries, which were missing because of our oversight.

Other relevant details are also edited to ensure clarity and eliminate contradictions, if any.

**Comment 4:** In line 255 also, the authors mentioned “Eight out of 17 countries have adopted the Beveridge model of healthcare………….” I wonder why the authors referred to 17 countries instead of 15??

**Response 4:** The response to comment 3 explains this.

**Comment 5:** The authors presented the comparison of NDSs with Glasgow Declaration and KAA of WHO’s Global Action Plan in the “discussion” section. I wonder if these are the main parts to be included in the “results” section. Also, the other findings of the results should have been presented in a concise manner.

**Response 5:** A section on comparison is now presented in a concise manner under the results section, along with a comparison matrix. The titles of these sub-sections in the discussion section are changed as:

Revised Title: “NDSs Clusters in the context of the Fundamental Rights of the Glasgow Declaration”

Original Title: “Comparison of NDSs Clusters with the Fundamental Rights of the Glasgow Declaration”, and

Revised Title: “NDSs Clusters in the context of the Key Action Areas of WHO’s Global Action Plan”

Original Title: “Comparison of NDSs Clusters with the Key Action Areas of WHO’s Global Action Plan”

The focus of these sub-sections is to highlight the intricacies of the fundamental rights / KAAs that are adhered, partially adhered or not adhered to and in what way there are adhered to, for each of the NDSs clusters, and which ones require attention in the NDSs. Also, the sub-section on “NDSs Clusters in the context of the Key Action Areas of WHO’s Global Action Plan” is substantially revised.

**Comment 6:** In the methods, the authors explained fiscal contextualization by examining different models. However, it is difficult to find any interrelation of financial implications of fundamental rights of Glasgow Declaration& KAA of WHO GAP with present study findings in the discussion section. Is this an additional area that the authors are looking into other than the objectives?

**Response 6:** In the context of the above two sections (renamed as explained in comment 5), which highlight the areas that need to be addressed by the NDSs, the interrelation of financial implications of fundamental rights of Glasgow Declaration& KAA of WHO GAP is explained in the discussion section. Also, the discussion on financial implications is shortened. A statement in conclusion section is also added to highlight its relevance.

Thank you once again for your time for meticulously going through our work, and your valuable suggestions to make our work more accessible and comprehensive for the prospective readers. We look forward to your response and to working with you through the review and publication process.

Sincerely,

**Smruti Bulsari**

Senior Research Officer, University of Essex (UNITED KINGDOM):

e-mail: [smruti.bulsari@essex.ac.uk](mailto:smruti.bulsari@essex.ac.uk)
